# Collective intelligence in medical decision-making: a systematic scoping review

**DOI:** 10.1186/s12911-019-0882-0

**Published:** 2019-08-09

**Authors:** Kate Radcliffe, Helena C. Lyson, Jill Barr-Walker, Urmimala Sarkar

**Affiliations:** 1 Center for Vulnerable Populations, University of California, San Francisco, USA; 2 Zuckerberg San Francisco General Hospital Library, University of California, San Francisco, San Francisco, CA USA

## Abstract

**Background:**

Collective intelligence, facilitated by information technology or manual techniques, refers to the collective insight of groups working on a task and has the potential to generate more accurate information or decisions than individuals can make alone. This concept is gaining traction in healthcare and has potential in enhancing diagnostic accuracy. We aim to characterize the current state of research with respect to collective intelligence in medical decision-making and describe a framework for diverse studies in this topic.

**Methods:**

For this systematic scoping review, we conducted a systematic search for published literature using PubMed, Embase, Web of Science, and CINAHL on August 8, 2017. We included studies that combined the insights of two or more medical experts to make decisions related to patient care. Studies that examined medical decisions such as diagnosis, treatment, and management in the context of an actual or theoretical patient case were included. We include studies of complex medical decision-making rather than identification of a visual finding, as in radiology or pathology. We differentiate between medical decisions, in which synthesis of multiple types of information is required over time, and studies of radiological scans or pathological specimens, in which objective identification of a visual finding is performed. Two reviewers performed article screening, data extraction, and final inclusion for analysis.

**Results:**

Of 3303 original articles, 15 were included. Each study examined the medical decisions of two or more individuals; however, studies were heterogeneous in their methods and outcomes. We present a framework to characterize these diverse studies, and future investigations, based on how they operationalize collective intelligence for medical decision-making: 1) how the initial decision task was completed (group vs. individual), 2) how opinions were synthesized (information technology vs. manual vs. in-person), and 3) the availability of collective intelligence to participants.

**Discussion:**

Collective intelligence in medical decision-making is gaining popularity to advance medical decision-making and holds promise to improve patient outcomes. However, heterogeneous methods and outcomes make it difficult to assess the utility of collective intelligence approaches across settings and studies. A better understanding of collective intelligence and its applications to medicine may improve medical decision-making.

**Electronic supplementary material:**

The online version of this article (10.1186/s12911-019-0882-0) contains supplementary material, which is available to authorized users.

## Background

Collective intelligence, in contrast to individual aptitude, is the ability of a group to perform a wide variety of tasks [1–3]. This concept can be referred to as “the wisdom of crowds,” and the classic example is Galton’s experiment in asking people with a range of expertise to look at a cow and estimate its weight [4]. He found that the average of all the estimates was correct within 1% of the actual weight, and the individual estimates were more likely to be incorrect [3, 4]. Studies have demonstrated that groups using collective intelligence in different cognitive tasks have high performance and can generate more accurate outcomes than the decisions of individuals alone [5–7]. Terms such as “collective intelligence,” “wisdom of the crowds,” and “crowdsourcing,” which are broad in scope, have been utilized to describe group decision-making in fields such as medicine, business, and ecology [3, 8, 9]. Groups can be comprised of either skilled experts, novices, or unskilled laypeople. “Crowdsourcing” typically refers to entrusting large, unskilled groups to complete tasks. In medicine, research has shown that crowdsourcing is an economical and efficient way to accurately accomplish work such as data or image processing and text interpretation [10, 11]. Similarly, research has shown that the use of multiple experts to classify radiological [5] or dermatological scans and specimens [12] is more accurate than individual assessments alone.

The ability of the group to outperform an individual on cognitive tasks has important implications for medical diagnosis and decision-making, given that team-based care has become a popular approach to the diagnostic process in delivering safer health care [13–16]. Activities that utilize the collective intelligence of medical experts have been part of a long-standing tradition: case conferences, expert consultation, and morning rounds are just a few examples of the conventional activities that depend upon the performance of groups. By harnessing the power of expert groups, collective intelligence provides an important opportunity to advance patient safety through improved medical decision-making and diagnosis.

Nevertheless, collective intelligence remains poorly characterized in the medical setting and its implications for expert medical decision-making lack clarity in the literature. Medical diagnosis and decision-making encompass a range of complexity and certainty. At one end of the spectrum, collective intelligence can be applied to objective identification of abnormalities on images, whether they are pathologic slides or radiologic scans, and recent research supports collective intelligence in these settings [5, 12]. In contrast, the diagnostic process in the clinical setting synthesizes subjective data, like clinical history and patient-reported information, with objective pathological and radiological findings, to continually generate new hypotheses [13]. Currently, little is known about collective intelligence in complex medical decision making, although early results with simulated cases are promising [17]. Recent research heralds the application of collective intelligence to radiology or pathology as proof that collective intelligence will improve accuracy across all medical specialties, yet there are no reviews of the application of collective intelligence in a typical diagnostic medical setting. Therefore, we conducted a systematic scoping review to both synthesize and characterize the current state of research on collective intelligence in medical decision-making.

Due to the relative novelty of the term “collective intelligence” in medicine, our review focuses on studies that describe efforts to make medical decisions through the combination of expert insights with an array of interventions. This review seeks to inform future studies that aggregate the insights of multiple individuals to improve patient care and safety. This review does not make inclusion or exclusion determinations based on the terminology used in the studies, because different studies employ terminology differently. For example, investigations may use terms such as “wisdom of the crowd,” “crowdsourcing,” or “collective insight,” to describe their work. We included the studies as long as they examine medical decision-making among medical professionals. For the purposes of this analysis, we utilize the term “collective intelligence” to describe interventions that utilize group insight to accomplish a task, with the understanding that such interventions may vary in methods and outcomes. We elected to examine application of collective intelligence methods to medical decision-making regardless of whether studies assessed decision or treatment accuracy. Due to the diverse nature of the research in this budding field, our review seeks to inform future studies by describing a framework to which future investigations may be applied.

## Methods

### Search strategy

We conducted a systematic scoping review to describe and analyze studies utilizing collective intelligence in medical decision-making. A systematic scoping review combines the rigorous nature of a systematic review, which seeks to answer an explicit scientific question, with a scoping review’s ability to synthesize heterogeneous research and establish the conceptual framework of a topic [18]. Because collective intelligence in medical decision-making is an emerging field with diverse research methods and outcomes, a systematic scoping review allowed us to characterize the broad state of the literature while maintaining a rigorous systematic search strategy.

Our systematic search strategy combined two concepts: collective intelligence and diagnosis or medical decision-making. We captured “collective intelligence” as a concept by including common, analogous concepts such as “crowdsourcing” and “wisdom of the crowd,” and broadening the search terms to include “collaborative” and “collective decision-making.” This strategy allowed us to identify literature broadly related to collective intelligence in medical decision-making absent a shared understanding of terms in the literature to characterize the concept. Given the incipient nature of this field, there are no prerequisite methodologies for generating collective intelligence in medicine. As such, we did not limit our search to specific methods for generating collective insight, such as case conferences or the use of computational rules (“majority”, “quorum”, and “weighted quorum”) in our search strategy. However, we included this literature if it met our search criteria.

We developed the search strategy in collaboration with a clinical librarian (JBW). Because of the lack of a shared definition of collective intelligence in the biomedical literature, we used a multi-step process to ensure the discovery and inclusion of a variety of terms to describe this concept. This search process entailed 1) identifying key terms from existing articles related to our topic, and 2) testing keywords and controlled vocabulary, including MeSH and Emtree terms, for each of the search concepts, using an iterative, collaborative approach with the entire research team. We developed the search in PubMed and applied to other databases accordingly. In accordance with National Academy of Sciences standards, the search strategy was peer reviewed by a second librarian using the Peer Review of Electronic Search Strategy (PRESS) Guideline [19]. We conducted the final search in PubMed, Embase, Web of Science, and CINAHL on August 8, 2017. Detailed search strategies for each database are located in Additional file 1. Handsearching of subject-specific journals included *Medical Decision Making, Diagnosis, BMC Medical Informatics and Decision Making,* and the Agency for Healthcare Research and Quality’s Patient Safety Network (PSNet) weekly literature review. We did not use grey literature because of our interest in research subjected to peer-review.

### Inclusion and exclusion criteria

Studies were included if they aggregated the medical opinions of at least two medical experts (physicians or trainees), with respect to specific clinical cases. We included studies in which participants examined real or simulated patient cases and made a judgement either collaboratively or individually. Because the diagnostic process involves complex medical decision-making before and after a diagnosis is made,[13] we included studies that utilize collective intelligence in any aspect of the medical decision-making process, including diagnosis, treatment, or management. Included studies make a judgment based on a specific, individual-level, actual or simulated patient case, rather than examining clinical syndromes in general (for example, expert opinions on hypertension guidelines). We did not limit our search to studies that detailed an analysis of the accuracy of collective intelligence. Due to our interest in characterizing the state of the literature surrounding collective intelligence, we did not limit the primary outcomes under investigation by included studies. Collective intelligence may be generated by a group of experts who make a collective decision, or may be the result of aggregation of the insights of multiple individuals. Therefore, even though these two processes differ in their methods and outcomes, both constitute collective intelligence in the current literature and both are included in this review.

We excluded studies in non-English languages, with no full-text, and those that did not include physicians or medical students. Studies that were secondary analyses of previously reported data were excluded due to our interest in primary data. Studies were also excluded if they assessed the opinions of radiologists and pathologists, or examined radiological scans or pathological specimens. Our goal was to uncover the utility of collective intelligence in diagnosis and decision-making, in which multiple sources of objective and subjective data generate a diagnosis over time, rather than in binary decisions such as identification or absence of a finding in radiology or pathology.

### Study selection

Two reviewers (KR & HCL) independently screened a random sample of 181 studies (10% of the overall total) by title and abstract and collaboratively reviewed screening decisions to ensure inter-rater consensus, in accordance with the current recommended standards for study selection [20, 21]. Two reviewers (KR & HCL) completed final screening for each article to determine inclusion and presented discrepancies to US for the final determination.

### Data extraction & critical appraisal

A standardized form was created to extract data in the following areas: 1) study setting, 2) study type and methodology, 3) characteristics of the intervention (e.g. intervention type, participant characteristics, and outcome measures used) and 4) results on primary outcomes as well as accuracy. Two reviewers (KR & HCL), with a third reviewer (US) available to resolve discrepancies, completed data extraction.

## Results

### Search results

**Fig. 1:**
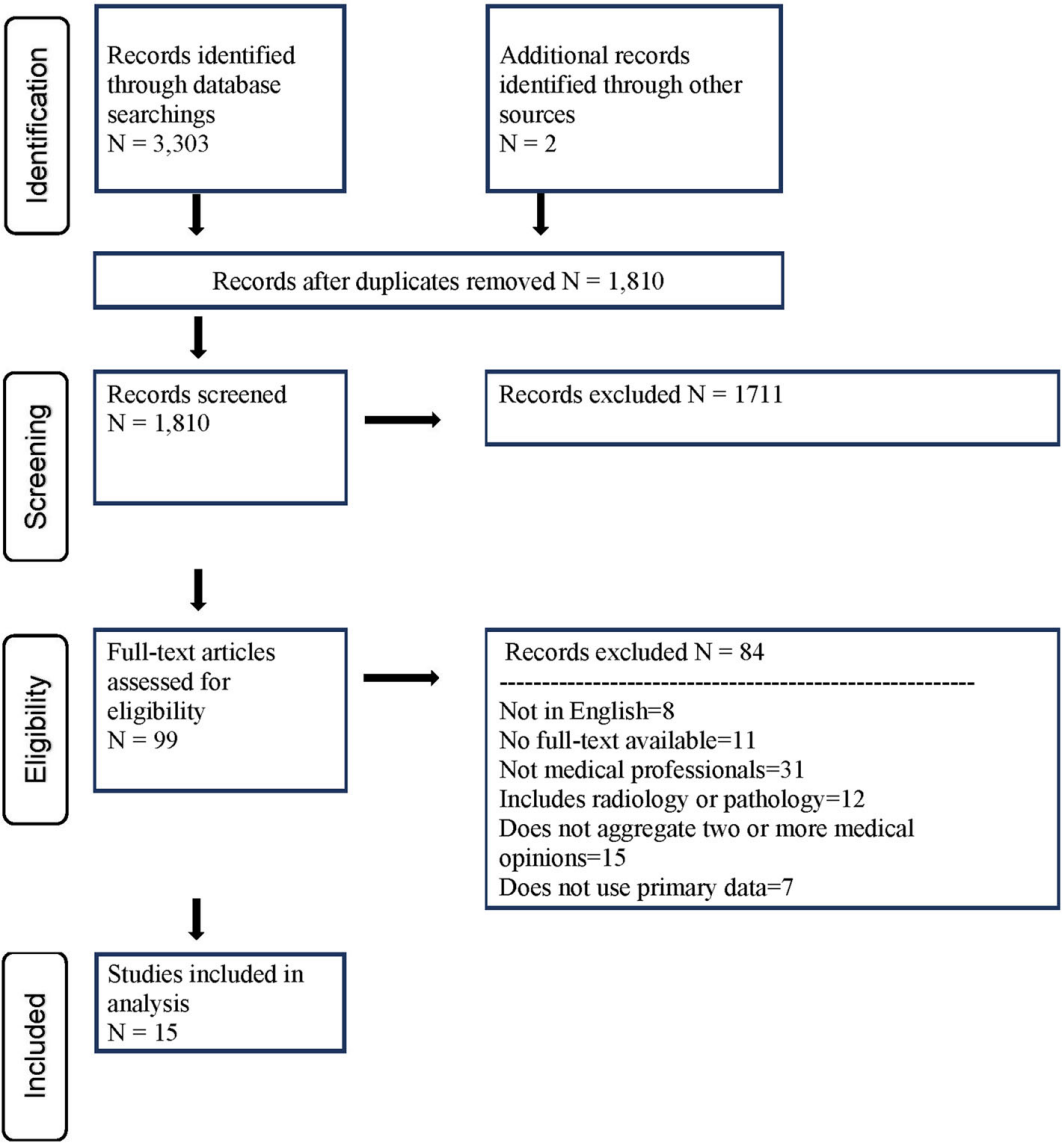
PRISMA chart

The literature search yielded 3303 articles and two additional articles after handsearching relevant journals. After excluding duplicates, we screened 1810 articles for inclusion based on title and abstract. The study team reviewed the full text of 99 articles and eliminated 84 based on previously established inclusion and exclusion criteria. Final analysis included 15 studies, as indicated by the PRISMA chart (Fig. 1) [22–36]. Characteristics of the included studies are presented below (Table 1).

**Table 1:** Collective intelligence study design

Study author, year	Description of experts	Real or simulated cases	Types of opinions aggregated	Study design	Relevance to collective intelligence
Gagliardi, 2007 [25]	20 general surgeons, 2 pathologists, 1 medical oncologist, 1 radiation oncologist	Real	Diagnosis, Treatment	Qualitative observational study to explore the role of multidisciplinary cancer conferences in practice	Describe collective output generated in multidisciplinary cancer conferences
Douzgou, 2016 [24]	Physicians with patients with malformation syndromes	Real	Diagnosis	Descriptive study of a consultation tool which generates collective insight	Assess a collective intelligence tool
Sternberg, 2017 [36]	International colleagues with urologic expertise	Real	Treatment	Use Twitter as a potential collective intelligence tool	Describe social media as a collective intelligence tool
Sims, 2014 [35]	Clinicians affiliated with academic departments: 28 from pediatrics, 27 from neurology, 10 from internal medicine, 4 from psychiatric, 11 from pediatric neurology, 5 others	Real	Diagnosis, Treatment	Descriptive study of a clinical consultation system which generates collective insight and qualitative evaluation of the tool	Describe a collective intelligence tool
Nault, 2009 [33]	5 spinal deformity surgeons	Real	Treatment	Feasibility study of a surgical decision-making tool as compared to a group of experienced surgeons	Compare collective intelligence generated by experts with a technology tool
Alby, 2015 [22]	1 oncologist and others from hematology, anesthesiology, surgery, and nephrology	Real	Diagnosis	Qualitative observational study of conversations about cancer cases between the chief oncologist and other physicians at a hospital	Characterize collective intelligence generated in usual practice
Kattan, 2013 [28]	24 urologists and oncologists	Real	Prognosis	Analysis of physician group accuracy as compared to a nomogram	Compare collective intelligence generated by experts with a technology tool
Kunina-Habenicht, 2015 [29]	283 medical students, 20 expert physicians	Real	Diagnosis	Descriptive study of the development of a computerized test to assess diagnostic accuracy; results were compared among medical students and expert physicians	Compare computer-generated collective intelligence of experienced physicians to medical students
Lajoie, 2012 [30]	14 third-year medical students	Simulated	Diagnosis, Treatment	Qualitative observational study of team discussions with or without a technology tool to aid collaboration	Optimize metacognitive activities in collective intelligence with a technology tool
Kalf, 1996 [27]	21 geriatricians, 21 geriatric-psychiatrists, 21 internists	Simulated	Diagnosis	Analysis of diagnoses generated by different specialties	Compare collective intelligence among different specialists
Larson, 1996 [31]	24 first-year interns, 24 residents, 24 medical students	Simulated	Diagnosis	Qualitative observational study of team diagnostic discussions when teams are exposed to different case information	Characterize collective intelligence generated when groups have different amounts of information about a case
Christensen, 2000 [23]	24 first year interns, 24 residents, 24 medical students	Simulated	Diagnosis	Qualitative observational study of team diagnostic discussions when given different amounts of shared and unshared information	Characterize collective intelligence generated when groups have different amounts of information about a case
Larson, 1998 [32]	48 interns and 24 third-year medical students	Simulated	Diagnosis	Qualitative observational study of team diagnostic discussions when teams are exposed to different case information and given instructions about sharing information	Characterize collective intelligence generated when groups have different amounts of information about a case
Semigran, 2016 [34]	234 physicians, including fellows and residents	Simulated	Diagnosis	Analysis of a collective intelligence tool as compared to the accuracy of symptom checker websites	Compare a collective intelligence tool to online symptom checkers
Hautz, 2015 [26]	88 medical students	Simulated	Diagnosis	Analysis of diagnostic accuracy when participants worked in pairs or individually	Compare collective intelligence of pairs to individual aptitude

### Participants and decisions in included studies

All 15 studies included medical experts contributing to the collective intelligence, including medical students, interns, residents, fellows, and attending physicians (Table 1), in accordance with our inclusion criteria. We did not include studies that employed laypeople’s input on medical decision-making. All studies included a minimum of two experts in the collective intelligence, with the maximum being 283 experts [34]. Although all included studies examined real (8/15 studies) or simulated (7/15 studies) patient cases, the types of cases or medical domains varied widely, including emergency medicine, urology, oncology, and others. We define “real cases” as those in which an existing patient provides the basis for the case, whereas “simulated cases” are developed by the study team and are not reflective of an actual patient. Due to our interest in any aspect of the medical decision-making process [13], we examined studies that generated a collective intelligence for a specific patient’s diagnosis, treatment, or prognosis. The majority of included studies utilized group insight to gather diagnoses (12/15), although some assessed treatment (5/15), prognosis (1/15), or a combination of each.

### Application of collective intelligence

**Fig. 2:**
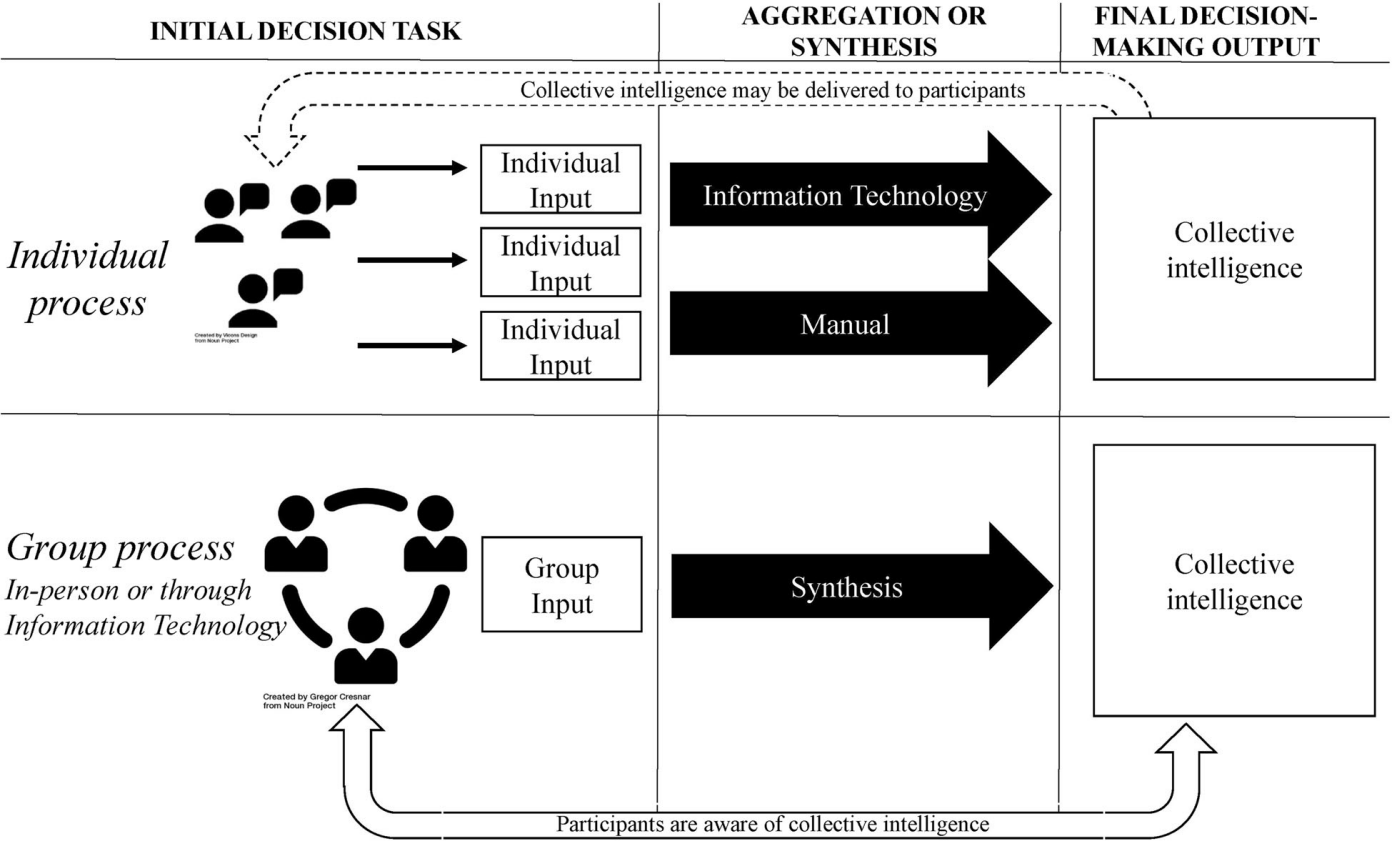
Collective intelligence framework

We developed a conceptual framework to characterize the different ways in which included studies conceptualized collective intelligence and medical decision-making. We identified three key aspects to the application of collective intelligence to complex medical decisions: 1) group versus individual cognition for the initial decision task, 2) how the collective intelligence synthesizes or aggregates initial decisions, and 3) the availability of the collective intelligence output to the study participants (Fig. 2). Despite the heterogeneous nature of the included studies, each of them applies a collective intelligence to complex medical decision-making. As the current medical literature describes a variety of interventions applying the concept of collective intelligence, this framework seeks to unify the field and clarify the elements that lead to generation of collective output based on the opinions of medical experts.

#### Initial decision task

In the included studies, a collective group output stems from an initial decision task completed by group or individual cognitive processes. Group processes involve open discussion among group members, in which experts contribute together to the development of the collective intelligence by way of discussion. Group processes of collective intelligence reflect conventional group activities in medicine, such as case conferences, in which a group discusses and comes to a consensus regarding a patient’s case. Recently, technology has enhanced the ability of individuals to collaborate as a group despite geographical distances, such as through the use of social media [36] to generate a consensus among geographically distant experts.

Individual processes, on the other hand, entail experts independently making judgements. Aggregation of these individual responses may require information technology to generate a collective intelligence, or may require manual efforts, such as “counting votes,” from the study team to synthesize a collective decision or output. Among the included studies, eight used group processes [22, 23, 25, 30–32, 35, 36], six used individual processes [24, 27–29, 33, 34], and one compared the two [26].

#### Method of aggregation

Next, both individual and group processes require an aggregation or synthesis of the insights of individuals or a group into a collective output. As indicated in Fig. 2, when a group jointly develops their initial decision, synthesis occurs as part of the initial input, as in a multidisciplinary case conference or by way of a virtual platform that utilizes information technology to leverage group discussion. In contrast, in individual processes of collective intelligence, manual or IT methods are required to generate a collective output from the individual inputs that experts contribute. A manual method may be as simple as averaging the numeric responses of each individual, as in the Galton example, or tallying dichotomous responses to arrive at a majority response. Sophisticated algorithms to synthesize individual opinions can also be applied using information technology.

IT facilitates collective intelligence by aggregating individual medical opinions through mobile or web-based technologies (individual processes) or by providing a virtual platform for expert discussion and consensus (group processes). Among the 15 studies included, seven generated a collective intelligence through the utilization of an IT platform [24, 26, 29, 33–36]. Individual processes aggregated by IT use the technology platform as a mechanism to collect individual opinions and synthesize them into the collective intelligence. In four of six individual-based studies, technology facilitated the aggregation of individual opinions into a collective intelligence [24, 29, 33, 34]. Among the eight group-based studies, aggregation of the individuals’ inputs happens at this initial stage by providing a web-based forum for discussion. Two studies used IT to facilitate a group process collective intelligence [35, 36], for example, by using the social media platform Twitter as a virtual platform for discussion [36]. In these studies, the IT platform serves as a forum for participants, who may be geographically dispersed, to discuss a clinical case and come to a group consensus. One study used both individual and group processes of collective intelligence facilitated by IT [29].

Individual processes of collective intelligence may also use manual methods to generate a collective intelligence from multiple, individual outputs. Participants provide their individual clinical assessment on paper and the study team collects each individual assessment and manually tallies them into a collective insight. Two of the included studies used manual aggregation [27, 28]. For example, one study presented participants with four patient cases and asked them to diagnose each case using a paper questionnaire. The study team then manually coded the responses of each of the 62 participants, grouping them into similar categories, and developing a collective intelligence for each case based on the majority rule [27].

In-person synthesis, on the other hand, occurs when real-time group discussion generates a consensus among participants. Activities such as case conferences or morning rounds that require participants to be present and discuss a case employ in-person synthesis. Of the eight studies that used group processes to generate collective intelligence, the majority (5/8) used in-person synthesis to generate a collective intelligence [22, 23, 25, 31, 32]. One study of the eight using group processes compared in-person and IT-enabled collective intelligence [30].

#### Availability of collective intelligence output

The final collective output has the potential to inform patient care decisions when it is available to participating individuals for review. For example, in group processes of collective intelligence (8 studies) [22, 23, 25, 30–32, 35, 36], the contributing group members are aware of the final results of consensus as they have participated in the consensus-making. On the other hand, through individual processes, the contributors may not be aware of the final decision-making output. In these cases, the study team or IT platform must decide whether to share the final consensus with the individuals. When contributors are aware of the final consensus, the collective intelligence results have the potential to impact future patient care. Our results show that the collective intelligence was available to participants in one of the six studies using individual processes [24], and in the study examining both group and individual processes [26]. For example, Douzgou et al. describe a procedure in which a final Expert Case Report, the product of collective intelligence, is routed back to the soliciting clinician. The availability of collective intelligence to clinicians will have important implications in whether or not collective intelligence can impact and improve patient care in real-time.

### Outcomes

All included studies examined some aspect of the collaborative decision-making process that generates a collective intelligence, but differ in their primary outcomes of interest (Table 2). In this review, the term collective intelligence describes the final decision-making output that is generated when experts contribute their collective insight to a case. Although the goals of collective intelligence were different among studies, each included study generated some form of group insight (collective intelligence) related to a real or simulated patient case.

**Table 2:** Study outcomes

Study author, year	Initial decision task process	Method of Aggregation /Synthesis	Study Outcomes	Results	Collective intelligence available to participants
Alby, 2015 [22]	Group	In-person	Extent of diagnostic uncertainty and perceived diagnostic complexity in discussions among experts	Study participants relied on three collaborative practices during informal conversations about cancer cases to organize the diagnostic decision-making process.	Yes
Christensen, 2000 [23]	Group	In-person	The ability of diagnostic teams to integrate shared and unshared case information into a differential diagnosis	Teams of study participants mentioned more shared than unshared information when diagnosing patient cases and were less likely to diagnosis a case accurately when team members had limited information. Experience of participants did not significantly impact diagnostic accuracy.	Yes
Gagliardi, 2007 [25]	Group	In-person	Extent to which multidisciplinary cancer conferences can address cancer-related information needs of clinicians	Multidisciplinary cancer conferences resolved cancer-related information needs, including treatment, diagnosis, pathology, and staging.	Yes
Larson, 1996 [31]	Group	In-person	The use and order of shared and unshared information in team diagnostic discussion and its contribution to diagnostic decision-making	Information that was known to all group members was more likely to be discussed than information unique to individuals. Team leaders performed an important function in ensuring quality group discussion and contributing to medical decision-making.	Yes
Larson, 1998 [32]	Group	In-person	Relation of shared and unshared information to diagnostic accuracy among teams	Shared case information was pooled more than unshared information among study participants. Diagnoses were more accurate when teams pooled more unshared information.	Yes
Sims, 2014 [35]	Group	Information technology	The utilization and user opinion of the crowdsourcing application in the clinical setting	A total of 170 consults were generated by 20 study participants, predominantly seeking assistance in medication use and complex decision-making from the crowd. Providers had a favorable opinion of using the tool in practice.	Yes
Sternberg, 2017 [36]	Group	Information technology	Extent to which Twitter can be used to share ideas about clinical case management	Twitter facilitated discussion among 11 participants from 5 countries that resulted in treatment suggestions.	Yes
Lajoie, 2012 [30]	Group	Information technology, in-person	Extent to which technology can enhance metacognitive activities in diagnostic discussion	Technology enabled more metacognitive activities in group discussion.	Yes
Douzgou, 2016 [24]	Individual	Information technology	The ability of a web-based service to generate clinical diagnosis for providers using an expert crowd and add value to practice.	The web-based service added value through the case report generated.	Yes
Kalf, 1996 [27]	Individual	Manual	Concordance in facts and diagnoses among different specialties examining clinical cases	Study participants differed systematically in the diagnoses they reached.	No
Kattan, 2013 [28]	Individual	Manual	Comparison of the accuracy of physician predictions with a nomogram	The nomogram was more accurate than physicians, regardless of medical specialty. There was variability among the decisions made by physicians.	No
Kunina-Habenicht, 2015 [29]	Individual	Information technology	Comparison of accuracy of diagnoses and time to diagnose between experts and medical students	Experts had higher accuracy rates and lower decision times than students. Diagnostic accuracy improved with year of study among students.	No
Nault, 2009 [33]	Individual	Information technology	Concordance between surgeons and a fuzzy logic model tool	Study participants made diagnostic decisions that were generally in agreement with decisions made by fuzzy logic model tool. There was large variability among the decisions made by study participants.	No
Semigran, 2016 [34]	Individual	Information technology	Comparison of the accuracy of differential diagnoses of physicians with online symptom checkers	Study participants listed the correct diagnosis first and within the top three diagnoses more often compared with symptom checkers. Study participants were more likely to list the correct diagnosis first for high-acuity vignettes and uncommon vignettes; symptom checkers were more likely to list the correct diagnosis first in low-acuity vignettes and common vignettes.	No
Hautz, 2015 [26]	Individual and Group	Information technology	Comparison of diagnostic performance of individuals with those working in pairs.	Pairs of study participants were more accurate and confident than individuals, but confidence was not dependent upon decision accuracy.	Yes/No

#### Group processes

Studies that employed group processes of collective intelligence often examined the group decision-making process, with primary outcomes such as metacognitive activities [30], information sharing [23, 31, 32], information needs [25], and insight into the complexity of the diagnostic process [22], but did not necessarily investigate the accuracy of collective intelligence or its benefit over individual decision-making. These studies explored the group decision-making process and contributed to our understanding of how group processes generate collective intelligence. For example, participants at a case conference were able to resolve their questions about a patient’s case by collaborating in a group process [25]. Such findings lend credence to the fact that generating collective insight among groups may improve patient care. Furthermore, studies interested in the role of information sharing and group consensus demonstrated that groups generate a poor collective intelligence when participating in unstructured group discussion after receiving different amounts of information [23, 31]; collective intelligence is improved when groups are instructed to share information [32].

Studies using group processes also examined various technology platforms to generate collective intelligence, such as Twitter as a forum for generating a group consensus [36] or a crowdsourcing application for electronic consultation [35]. Finally, one study demonstrated that a collaborative technology platform could enhance a group’s cognitive skills [30]. While these studies may seem overly simplistic compared to the sophistication of recent automated methods or collective intelligence platforms, they are practical applications of “wisdom of the crowds” to complex medical decision-making and are relatable applications of collective intelligence to usual practice.

#### Individual processes

Conversely, studies that utilized individual processes of collective intelligence examined the utility of collective intelligence technology platforms, such as the Dysmorphology Diagnostic System, which allowed physicians to seek diagnostic input from others and generated a collective intelligence case report for the consulting physicians [24]. Included studies also compared the collective insight of medical experts to other automated methods of generating diagnosis, for example, comparing expert surgeons to an automated surgical decision model tool [33], a nomogram [28], or comparing physicians to online symptom checker websites [34]. These studies compared the collective intelligence of experts to an existing tool to uncover the utility of collective insight as compared to sophisticated automated methods. While they did not compare groups of physicians to an individual decision-maker, they present important findings as medicine increasingly relies on tools such as technology and the internet.

Studies that utilized individual processes of collective intelligence also compared the diagnostic accuracy of different physicians groups [27], finding that specialists varied systematically in the diagnoses they reached, and that experts were more accurate than medical students [29]. These findings imply that while collective intelligence may be a useful tool in diagnosis, it is important to consider the level of expertise and specialty of expert participants generating the collective intelligence. Finally, one study compared the diagnostic accuracy of medical student pairs with individual medical students and found that pairs were more accurate in their diagnoses [26].

#### Diagnostic accuracy

Diagnostic accuracy, or the ability of the group to determine the correct diagnosis in simulated or real cases with known correct responses, was an outcome in six of the fifteen studies [23, 26, 28, 29, 31, 34]. In these studies, physician groups were shown to be more accurate in making a diagnosis when complete information was provided to them as opposed to hidden or incomplete information [23, 31], and when physicians were prompted to pool all their information before making a determination [32]. These results provide important information about strategies by which to facilitate generation of an accurate collective output. Additionally, when compared to online automated symptom checkers, physicians had better diagnostic accuracy [34]. As compared to novices, expert physicians had better diagnostic accuracy and faster decision times [29], and novice pairs were more accurate than those working alone [26]. This finding has implications in future investigations, which may choose to use the combined insights of experts rather than novices, such as students, as participants in collective intelligence.

## Discussion

In this systematic scoping review, we identified 15 studies that describe the use of collective intelligence in medical decision-making. Although included studies vary in their interventions and outcomes, their examination of collective intelligence processes demonstrates the potential for collective intelligence, or group insight, to impact medical decision-making. In particular, studies included in our review reveal that collective intelligence IT platforms can allow physicians to resolve uncertainty in diagnosis and treatment decisions [24, 35], can be more accurate than online symptom checkers [34], and can facilitate group processes of collective intelligence [36] to improve metacognitive activities and collective insight [30]. The use of technology as a means of aggregating multiple opinions into a collective intelligence has important implications for improving patient care. Technology connects individuals that are separated geographically, and can be an important tool to connect physicians with the expertise of others [37]. Therefore, the combination of collective intelligence, which has the potential to improve diagnostic accuracy, with the expanding reliance on technology in medicine, has the potential to lead to improved patient care when implemented into practice.

Beyond the aggregation of multiple opinions into the collective intelligence, a critical component to improving patient care is the availability of the collective intelligence to study participants. When the collective insight of experts is available to them, participants are able to subsequently make decisions that impact patient care, particularly when real cases are used for analysis. Five of the fifteen included studies generated collective intelligence based on real cases and allowed participants to review the collective intelligence output [22, 24, 25, 36]; however, no studies described the patient care effects of the collective intelligence when it is made available to participants. In order to assess the extent to which collective intelligence can improve actual patient outcomes, it is imperative that researchers investigate and report implications for patient outcomes in their work.

In medical practice, long-standing activities such as case conferences, specialist consultation, and tumor boards have attempted to create collective intelligence without the involvement of technology tools. While these activities may lack the sophistication of technology-enabled methods, they are part of usual care for many physicians. Future studies that examine collective intelligence should bear in mind that similar activities already exist in practice and that technology or artificial intelligence can possibly optimize these processes, but must consider physician workflows in clinical care. Moreover, studies should consider using real cases rather than simulated cases in future research to better understand the short- and long-term ramifications of reliance on collective intelligence.

### Limitations

Our review included only English-language publications. We did not limit the outcomes or interventions of studies in order to keep the review scoping in nature. Due to the heterogeneity among studies using collective intelligence, as well as the diverse and dynamic range of terms used to describe this phenomenon, it is possible that some relevant articles were not included.

## Conclusion

This systematic scoping review is the first to our knowledge to characterize collective intelligence in medical decision-making. Our review describes collective intelligence that is generated by medical experts and distinct from terms such as “crowdsourcing” that do not use experts to make medical judgments. All included studies examine collective intelligence as it pertains to specific cases, rather than simply describing collaborative decision-making or other decision aids. In this review we present a novel framework to describe investigations into collective intelligence. Studies examined two distinct forms of the initial decision task in collective intelligence: individual processes that were subsequently aggregated, versus group synthesis in which the diagnostic thinking was initiated in a group setting. The initial decision task is followed by aggregation or synthesis of opinions to generate the collective decision-making output. When a group jointly develops their initial decision, synthesis occurs as part of the initial input, whereas in individual processes, manual or IT methods are required to generate a collective output from the individual inputs that experts contribute. The final collective output can then be routed back to the decision-makers to potentially influence patient care. The impact of these approaches on patient outcomes remains unclear and merits further study. Similarly, further research is needed to determine how to best incorporate these approaches into clinical practice.

## Additional file

Additional file 1: Search strategies for published literature. (DOCX 15 kb)

## Data Availability

The search conducted in this article is available in the Additional file 1 and from the corresponding author upon request.

## Abbreviations

HCL: Helena C. Lyson
IT: Information technology
JBW: Jill Barr-Walker
KR: Kate Radcliffe
US: Urmimala Sarkar

## References

[1] Woolley AW, Chabris CF, Pentland A (2010). Evidence for a collective intelligence factor in the performance of human groups. Science.

[2] Krause J, Ruxton GD, Krause S (2010). Swarm intelligence in animals and humans. Trends Ecol Evol.

[3] Surowiecki J (2004). The wisdom of crowds: why the many are smarter than the few and how collective wisdom shapes business, economies, societies, and nations.

[4] Galton F (1907). Vox Populi. Nature.

[5] Wolf M, Krause J, Carney PA (2015). Collective intelligence meets medical decision-making: the collective outperforms the best radiologist. PLoS One.

[6] Toyokawa W, Kim HR, Kameda T (2014). Human collective intelligence under dual exploration-exploitation dilemmas. PLoS One.

[7] Kurvers RH, Wolf M, Naguib M (2015). Self-organized flexible leadership promotes collective intelligence in human groups. R Soc Open Sci.

[8] Hernandez-Chan GS, Ceh-Varela EE, Sanchez-Cervantes JL (2016). Collective intelligence in medical diagnosis systems: a case study. Comput Biol Med.

[9] Sole R, Amor DR, Duran-Nebreda S (2016). Synthetic collective intelligence. Biosystems.

[10] Créquit P, Mansouri G, Benchoufi M (2018). Mapping of crowdsourcing in health: systematic review. J Med Internet Res.

[11] Wang X, Mudie L, Brady CJ (2016). Crowdsourcing: an overview and applications to ophthalmology. Curr Opin Ophthalmol.

[12] Kurvers RH, Krause J, Argenziano G (2015). Detection accuracy of collective intelligence assessments for skin Cancer diagnosis. JAMA Dermatol.

[13] Ball JR, Balogh E (2016). Improving diagnosis in health care: highlights of a report from the National Academies of sciences, engineering, and medicine. Ann Intern Med.

[14] Institute of Medicine Committee on Quality of Health Care in A. In: Kohn LT, Corrigan JM, Donaldson MS, eds. To Err is human: building a safer health system. Washington (DC): National Academies Press (US) Copyright 2000 by the National Academy of Sciences. All rights reserved. 2000. 25077248

[15] Greiner AC, Knebel E, Institute of Medicine Committee on the Health Professions Education S (2003). Health professions education: a bridge to quality.

[16] Bunting RF, Groszkruger DP (2016). From to err is human to improving diagnosis in health care: the risk management perspective. J Healthc Risk Manag.

[17] Barnett ML, Boddupalli D, Nundy S (2019). Comparative accuracy of diagnosis by collective intelligence of multiple physicians vs individual physicians. JAMA Netw Open.

[18] Peters MD, Godfrey CM, Khalil H (2015). Guidance for conducting systematic scoping reviews. Int J Evid Based Healthc.

[19] Institute of Medicine Committee on Standards for Systematic Reviews of Comparative Effectiveness R. In: Eden J, Levit L, Berg A, et al., eds. Finding what works in health care: standards for systematic reviews. Washington (DC): National Academies Press (US) copyright 2011 by the National Academy of Sciences. All rights reserved. 2011. 24983062

[20] Institute of Medicine (US) Committee on Standards for Systematic Reviews of Comparative Effectiveness Research; Eden J, Levit L, Berg A, Morton S, editors. Finding what works in health care: standards for systematic reviews. Washington (DC): National Academies Press (US); 2011. 24983062

[21] Umscheid CA (2013). A primer on performing systematic reviews and meta-analyses. Clin Infect Dis.

[22] Alby F (2015). Zucchermaglio, et al. diagnostic decision making in oncology: creating shared knowledge and managing complexity. Mind Culture Activity.

[23] Christensen C, Larson (2000). Decision making of clinical teams: communication patterns and diagnostic error. Med Decis Mak.

[24] Douzgou S, Pollalis (2016). Collaborative crowdsourcing for the diagnosis of rare genetic syndromes: the DYSCERNE experience. Public Health Genomics.

[25] Gagliardi AR, Wright (2007). The role of collegial interaction in continuing professional development. J Contin Educ Heal Prof.

[26] Hautz Wolf E., Kämmer Juliane E., Schauber Stefan K., Spies Claudia D., Gaissmaier Wolfgang (2015). Diagnostic Performance by Medical Students Working Individually or in Teams. JAMA.

[27] Kalf AJ, Spruijt M (1996). Variation in diagnoses: influence of specialists' training on selecting and ranking relevant information in geriatric case vignettes. Soc Sci Med.

[28] Kattan MW, Yu C, Stephenson AJ (2013). Clinicians versus nomogram: predicting future technetium-99m bone scan positivity in patients with rising prostate-specific antigen after radical prostatectomy for prostate Cancer. Urology.

[29] Kunina-Habenicht Olga, Hautz Wolf E., Knigge Michel, Spies Claudia, Ahlers Olaf (2015). Assessing clinical reasoning (ASCLIRE): Instrument development and validation. Advances in Health Sciences Education.

[30] Lajoie SP, Lu (2012). Supporting collaboration with technology: does shared cognition lead to co-regulation in medicine?. Metacogn Learn.

[31] Larson JR (1996). Diagnosing groups: charting the flow of information in medical decision-making teams. J Pers Soc Psychol.

[32] Larson JR (1998). Diagnosing groups: the pooling, management, and impact of shared and unshared case information in team-based medical decision making. J Pers Soc Psychol.

[33] Nault ML, Labelle (2009). Fuzzy-logic-assisted surgical planning in adolescent idiopathic scoliosis. J Spinal Disord Tech.

[34] Semigran Hannah L., Levine David M., Nundy Shantanu, Mehrotra Ateev (2016). Comparison of Physician and Computer Diagnostic Accuracy. JAMA Internal Medicine.

[35] Sims MH, Bigham (2014). Crowdsourcing medical expertise in near real time. J Hosp Med.

[36] Sternberg Kevan M, Loeb Stacy L, Canes David, Donnelly Laura, Tsai Mitchell H (2017). The use of Twitter to facilitate sharing of clinical expertise in urology. Journal of the American Medical Informatics Association.

[37] Liddy C, Drosinis P, Keely E (2016). Electronic consultation systems: worldwide prevalence and their impact on patient care—a systematic review. Fam Pract.

