# Supplementary material for: Collective intelligence in medical decision-making: a systematic scoping review
Source: BMC Med Inform Decis Mak. 2019 Aug 9;19:158. doi: 10.1186/s12911-019-0882-0 (PMC6688241; doi:10.1186/s12911-019-0882-0)
Supplement: Supplementary file 1 — Search strategies for published literature. (DOCX 15 kb) [file 12911_2019_882_MOESM1_ESM.docx]

**Additional file 1.** Search strategies for published literature. All searches were conducted on August 8, 2017.

| **Database** | **Search strategy** | **Number of results** |
| --- | --- | --- |
| PubMed | (("collective intelligence"[tw] OR "Crowdsourcing"[Mesh] OR "crowdsourcing"[tw] OR crowdsourced[tw] OR crowdsource[tw] OR "wisdom of the crowd"[tw] OR "wisdom of crowds"[tw] OR "collective analysis"[tw] OR "collective decision"[tw] OR "collective decisions"[tw] OR "collaborative decision"[tw] OR "collaborative decisions"[tw] OR "double review"[tw] OR "double reviewed"[tw] OR "double interpreted"[tw] OR "double interpretation"[tw] OR "double read"[tw] OR "double reads"[tw] OR "double reader"[tw] OR "double readers"[tw] OR "double reading"[tw] OR "double reporting"[tw] OR "shared knowledge"[tw] OR "shared wisdom"[tw] OR "shared information"[tw] OR "collaborative decision"[tw])  AND  (diagnosis[tw] OR diagnostic[tw] OR diagnostics[tw] OR diagnose[tw] OR diagnosed[tw] OR diagnoses[tw] OR misdiagnose[tw] OR misdiagnosed[tw] OR misdiagnosis[tw] OR misdiagnoses[tw] OR decision-making[tw] OR "decision making"[tw] OR "Clinical Decision-Making"[Mesh] OR "Decision Making, Computer-Assisted"[Mesh] OR "Decision Support Systems, Clinical"[Mesh] OR "Diagnosis"[Majr] OR "Diagnosis, Computer-Assisted"[Mesh])) | 1126 |
| Embase | ("collective intelligence" OR 'crowdsourcing'/exp OR crowdsourcing OR crowdsourced OR crowdsource OR "wisdom of the crowds" OR "wisdom of the crowd" OR "wisdom of crowd" OR "wisdom of crowds" OR "collective analysis" OR "collective decision" OR "collective decisions" OR "double review" OR "double reviews" OR "double reviewed" OR "double reviewer" OR "double reviewing" OR "double interpret" OR "double interprets" OR "double interpreted" OR "double interpreter" OR "double interpreting" OR "double interpretation" OR "double read" OR "double reads" OR "double reader" OR "double readers" OR "double reading" OR "double reporting" OR "shared knowledge" OR "shared wisdom" OR "shared information" OR "collaborative decision")  AND  (diagnosis OR diagnostic OR diagnostics OR diagnose OR diagnosed OR diagnoses OR misdiagnose OR misdiagnosed OR misdiagnoses OR misdiagnosis OR decision-making OR "decision making" OR 'medical decision making'/exp OR 'clinical decision making'/exp OR 'clinical decision support system'/exp OR 'diagnosis'/mj) | 1308 |
| Web of Science | ("collective intelligence" OR crowdsourcing OR crowdsourced OR crowdsource OR "wisdom of the crowds" OR "wisdom of the crowd" OR "wisdom of crowd" OR "wisdom of crowds" OR "collective analysis" OR "collective decision" OR "collective decisions" OR "double review" OR "double reviews" OR "double reviewed" OR "double reviewer" OR "double reviewing" OR "double interpret" OR "double interprets" OR "double interpreted" OR "double interpreter" OR "double interpreting" OR "double interpretation" OR "double read" OR "double reads" OR "double reader" OR "double readers" OR "double reading" OR "double reporting" OR "shared knowledge" OR "shared wisdom" OR "shared information" OR "collaborative decision")  AND  (diagnosis OR diagnostic OR diagnostics OR diagnose OR diagnosed OR diagnoses OR misdiagnose OR misdiagnoses OR misdiagnose OR misdiagnosed OR misdiagnoses OR misdiagnosis OR decision-making OR "decision making" OR "clinical decision support system")  AND   (health OR medicine OR medical OR clinical OR healthcare OR physician* OR practitioner* OR nurs* OR doctor* OR provider*) | 527 |
| CINAHL | ("collective intelligence" OR crowdsourcing OR crowdsourced OR crowdsource OR (MH "Crowdsourcing") OR "wisdom of the crowds" OR "wisdom of the crowd" OR "wisdom of crowd" OR "wisdom of crowds" OR "collective analysis" OR "collective decision" OR "collective decisions" OR "double review" OR "double reviews" OR "double reviewed" OR "double reviewer" OR "double reviewing" OR "double interpret" OR "double interprets" OR "double interpreted" OR "double interpreter" OR "double interpreting" OR "double interpretation" OR "double read" OR "double reads" OR "double reader" OR "double readers" OR "double reading" OR "double reporting" OR "shared knowledge" OR "shared wisdom" OR "shared information" OR "collaborative decision")  AND  (diagnosis OR diagnostic OR diagnostics OR diagnose OR diagnosed OR diagnoses OR misdiagnose OR misdiagnoses OR misdiagnose OR misdiagnosed OR misdiagnoses OR misdiagnosis OR (MH "Diagnosis") OR (MH "Decision Making, Clinical") OR (MH "Decision Support Systems, Clinical") OR decision-making OR "decision making" OR "clinical decision support system") | 342 |
| Total number of results |  | 3303 |
| Total number of duplicates |  | 1493 |
| **Total number after de-duplication** |  | **1810** |
